# News from around the RNA world: new avenues in RNA biology, biotechnology and therapeutics from the 2022 SIBBM meeting

**DOI:** 10.1242/bio.059597

**Published:** 2022-10-14

**Authors:** Virginia Brancato, Ilaria Brentari, Lucia Coscujuela Tarrero, Mattia Furlan, Francesco Nicassio, Michela A. Denti

**Affiliations:** ^1^ Center for Genomic Science IIT@SEMM, Italian Institute of Technology, Milan 20139, Italy; ^2^ Department of Cellular, Computational and Integrative Biology - CIBIO, University of Trento, Trento 38123, Italy

**Keywords:** Non-coding RNAs, microRNAs, Circular RNAs, RNA therapies

## Abstract

Since the formalization of the Central Dogma of molecular biology, the relevance of RNA in modulating the flow of information from DNA to proteins has been clear. More recently, the discovery of a vast set of non-coding transcripts involved in crucial aspects of cellular biology has renewed the enthusiasm of the RNA community. Moreover, the remarkable impact of RNA therapies in facing the COVID19 pandemics has bolstered interest in the translational opportunities provided by this incredible molecule. For all these reasons, the Italian Society of Biophysics and Molecular Biology (SIBBM) decided to dedicate its 17th yearly meeting, held in June 2022 in Rome, to the many fascinating aspects of RNA biology. More than thirty national and international speakers covered the properties, modes of action and applications of RNA, from its role in the control of development and cell differentiation to its involvement in disease. Here, we summarize the scientific content of the conference, highlighting the take-home message of each presentation, and we stress the directions the community is currently exploring to push forward our comprehension of the RNA World 3.0.

## Teaser

**Summary:** Review of the Italian Society of Biophysics and Molecular Biology (SIBBM) 17th yearly meeting, held in June 2022 in Rome, dedicated to the many fascinating aspects of RNA biology.

## Introduction

The role played by RNA in regulating the information flow from DNA to proteins was formalized in 1956 with the formulation of the Central Dogma of Molecular Biology (Crick, 1958). However, the work of Professor Crick did not deny the existence of interactions between RNA molecules and either other transcripts or DNA, and indirectly supported a broader vision of gene expression (Crick, 1970). This perspective partially anticipated the revolution represented by the discovery that RNA molecules may have regulatory functions besides the protein-coding potential, participating to complex mechanisms deeply involved in the regulation of numerous biological processes, from physiology to pathology (Statello et al., 2021).

Genetic information is transferred from DNA to RNA to protein, where RNA has been considered just an intermediary between DNA and proteins. The ‘RNA world’ became more and more complex in the latest 20 years with the discovery of non-coding RNAs, which have great importance in cellular processes. However, the border in the definition of coding and non-coding RNAs is quite blurry, since the two categories are interdependent. The two main functions of RNA, translation and structural role, could be associated to the different sub-cellular localization of the RNAs, since the translation takes place in the cytoplasm while the non-coding/regulatory functions are mainly exerted in the nucleus. Moreover, RNA molecules undergo alternative splicing, a process that could generate coding and non-coding isoforms from the same loci (Hubé and Francastel, 2018). New technologies allowed the understanding of the regulatory role of RNAs in development and disease. In fact, some mRNA transcripts, besides their coding potential, also act as regulatory RNAs through their secondary structure; while some lncRNAs may encode small peptides and, hence, modulate cellular activities (Li and Liu, 2019).

**Fig. 1.**
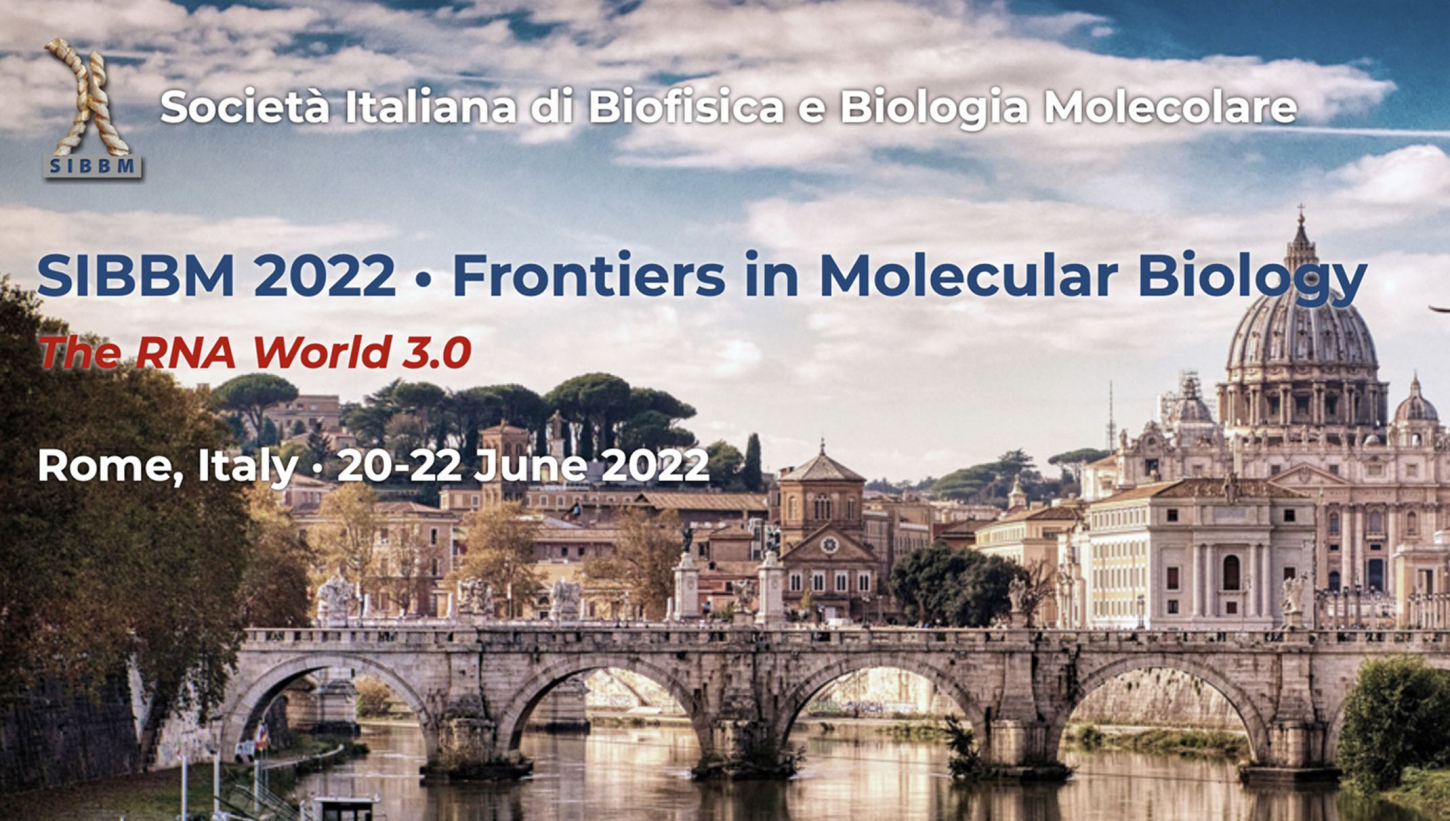
The opening of the web page of the SIBBM 2022 meeting (sibbm2022.azuleon.org).

For this reason, the Italian Society of Biophysics and Molecular Biology (SIBBM) decided to dedicate its 17th yearly meeting, held in June 2022 in Rome, to this fascinating aspect of RNA (Fig. 1). The Meeting was organized by Irene Bozzoni, Stefano Gustincich, Francesco Nicassio and Michela A. Denti. More than thirty speakers, both invited and selected from the presented abstracts, covered the biology, properties, modes of action and applications of RNA, from its roles in the control of development and cell differentiation to its involvement in disease.

In this Meeting Review, we report on the current trends in RNA biology, focusing on their regulatory functions provided by (i) the structure of RNAs, (ii) their cellular localisation and (iii) the interactions with RNA binding proteins. An overview of the non-coding RNA and their implications with epigenomics regulation is also highlighted, focusing on circRNAs, lncRNAs and miRNAs. The SIBBM meeting drew attention to the newest technologies for RNA sequencing and analysis, such as direct sequencing, reserving a section of the meeting for describing state-of-the-art technology in this field.

The conference welcomed about 270 participants, half of them junior researchers and graduate students. Here, we summarize the scientific content of the conference, highlighting the take-home message of each presentation, and we stress the directions which the community is exploring to push forward our comprehension of the RNA World 3.0.

## RNA structure, localization and RNA–protein interaction

The first section of the conference, RNA structure, localization and RNA-protein interaction, was chaired by Valeria Poli and Francesco Nicassio. From the contributions of the speakers, a broad interconnection between non-coding RNAs (ncRNAs) and RNA binding proteins (RBPs) emerged. In this regard, the recently discovered concept of phase separation been particularly relevant across different biological contexts, with a particular impact on neuronal biology. RNA–protein interactions appeared to be modulated according to the structures (both primary and secondary) of the transcripts and emerged as a major factor driving RNA and protein localization.

The first invited speaker, **Fabrizio d'Adda di Fagagna** (IFOM – Milan, Italy), focused on the role played by ncRNAs in the DNA damage response, highlighting the contribution of his group to dissecting this mechanism. He presented a mechanism based on the synthesis and processing of long non-coding RNAs (lncRNA) at double-strand break sites. Through phase separation, the resulting transcripts mediate the recruitment and localization of proteins involved in DNA repair. Interestingly, since these RNAs are site-specific, their inhibition by antisense oligonucleotides (ASOs) allows the impairment of DNA damage signaling and repair at specific loci of the genome, providing an excellent resolution to investigate this process (D'Alessandro and d'Adda di Fagagna, 2018; Francia et al., 2012; Michelini et al., 2017). The work of d'Adda di Fagagna found a natural application in the study of telomeres, where DNA damage accumulates over time, and disclosed a relevant regulatory layer involved in physiological and pathological aging (Aguado et al., 2019; Rossiello et al., 2017).

The section continued with the contribute of **Marie Laure Baudet** (University of Trento – Trento, Italy), who held the Armenise-Harvard Foundation Lecture, and whose research group is studying RNA localization. Specifically, the Baudet Lab used molecular beacon technology and advanced imaging approaches to characterize the transport of pre-miR-181a-1 in neurons’ compartments, suggesting that pre-miRNAs are shipped to distal axons by hitchhiking on late endosomes/lysosomes. In the growth cone central domain, where they are stored, pre-miRNAs are processed into newly generated mature miRNAs (miR-181a-5p, miR-181a-3p) in response to Semaphorin 3A. The mature miRNAs regulate the local translation of TUBB3, and impact axons’ growth and collapse. Overall, the results presented by Dr. Baudet not only reveal critical insight into the molecular mechanisms of ncRNA subcellular localization, but also provide fundamental knowledge of axonal transport; a process deeply involved in several neurodegenerative disorders (Corradi and Baudet, 2020; Corradi et al., 2020).

**Eleonora D'Ambra** (Italian Institute of Technology and Sapienza University – Rome, Italy) stayed on the subject, talking about the application of Basescope-FISH assays to study ncRNA localization in motoneurones. D'Ambra focused on the motoneurone-specific circular RNA (circRNA) circ-Hdgfrp3, which was found to traffic along neurites during cell maturation. This behaviour is impaired by oxidative stress, which causes a partial circ-Hdgfrp3 retention into stress granules. In motoneurones carrying FUS mutation, a condition linked to amyotrophic lateral sclerosis (ALS), the same stimulus results in the great segregation of circ-Hdgfrp3 into cytoplasmic mutant FUS assemblies. Interestingly, upon stress removal, the circular RNA is quickly released from stress granules but remains trapped into FUS-inclusions with the consequent impairment of its trafficking and function (D'Ambra et al., 2021).

Neuronal development was also the focus of the contribute by **Damiano Mangoni** (Italian Institute of Technology – Genoa, Italy) who investigated the involvement of transposable elements Long Interspersed Nuclear Elements-1 (L1) in corticogenesis. L1-derived RNAs are required for regulating the balance between proliferation and differentiation in progenitor cells, and to establish the transcriptional program leading to neuronal differentiation and maturation. The data presented by Mangoni suggest an interaction of L1 RNAs with the Polycomb Repressive Complex 2 (PRC-2) to regulate the deposition of the repressive epigenetic mark H3K27me3 on PRC-2-target genes. This study position L1 RNAs as crucial signaling hubs for genome-wide chromatin remodeling, enabling the fine-tuning of gene expression during brain development.

The next speaker, **Gian Gaetano Tartaglia** (IIT and Sapienza University – Rome, Italy), was invited to present the work of his group on RNA-mediated phase separation. First, he presented novel algorithms able to integrate local properties of protein and RNA structures to quantitatively predict long RNA binding proteins partners. These technologies enabled the Tartaglia Lab to reveal the association of ncRNAs (e.g. Xist) with proteins involved in the regulation of transcriptional and translational programs, as well as neurodevelopmental (FXTAS) and neurodegenerative diseases (Parkinson's disease) (Cid-Samper et al., 2018; Cirillo et al., 2017; Marchese et al., 2017). Furthermore, these studies highlighted features of RNAs, like the extent of double-stranded regions, which control the number of possible RBPs. This has relevant consequences in the regulation of phase separation events, and in viral infection biology. In this regard, the 5′ end of SARS-CoV-2 was predicted to be highly structured and, for this reason, able to interact with several human proteins that promote viral replication (Bolognesi et al., 2016; Cerase et al., 2019; Sanchez de Groot et al., 2019; Vandelli et al., 2020).

The following speaker, **Mihaela Zavolan** (University of Basel – Basel, Switzerland), was invited to give the Human Technopole Lecture. She presented the work of her group on 3′ untranslated regions (3'UTRs) and alternative polyadenylation (APA). She gave an overview of the methodologies and resources developed by her lab to profile polyadenylation signals and 3'UTR isoforms, as well as RBP regulators of 3′ end processing. Moreover, she presented very interesting applications of her methodologies to the investigation of APA in cancer. For instance, she introduced approaches to infer poly(A) sites usage (PAQR) and motifs responsible for this regulation (KAPAC), showing their application to identify a key regulator of APA in glioblastoma (PTBP1). She also presented a method (SCUREL) suitable to quantify changes in 3′ UTR length between groups of cells. This tool was applied to investigate the differential alternative polyadenylation between malignant and infiltrated cells in lung adenocarcinoma, and it revealed a general trend toward 3′ UTR shortening for cells in the tumor microenvironment (not only malignant ones) versus normal counterparts. In general, these techniques proved to be extremely informative to investigate 3'UTR isoforms, with potential repercussions on several human pathologies including cancer and haematological, immunological and neurological diseases (Burri and Zavolan, 2021; Gruber and Zavolan, 2019; Gruber et al., 2018; Herrmann et al., 2019).

**Marianna Maniaci** (PhD candidate at the European Institute of Oncology – Milan, Italy) presented her study on the arginine methylation of RNA binding proteins. She profiled RBPs–RNA interaction through Orthogonal Organic Phase Separation after the pharmacological inhibition of protein arginine methyltransferases (PRMTs), and during cisplatin-induced replicative stress. Indeed, even if previous studies from her group have demonstrated that cisplatin treatment could change PRMT subcellular localization, her results suggest a novel layer of complexity. In response to the cisplatin treatment the R-hypomethylation on RBPs, the consequent modulation of RBP-RNA binding and their subcellular localization can occur (Maniaci et al., 2021).

**Giorgio Giardina** (Sapienza University – Rome, Italy) presented an example of riboregulation describing his work on the structural characterization of the complex between cytosolic serine hydroxymethyltransferase and its cognate RNA modulator. These are the first 3D structural data of a riboregulated enzyme. His results suggest that the RNA molecule acts as a conformational switch, allosterically regulating the enzyme and locking it in a conformation unfavorable for the binding of the substrate serine. This observation provides insights on how RNA can allosterically control the activity of metabolic enzymes, and it offers an approach for future RNA-mediated therapies based on the targeting of these enzymes (Guiducci et al., 2019; Monti et al., 2021).

Finally, **Alessandro Rosa** (Sapienza University – Rome, Italy) presented the work on motoneuronal phenotypes upon ALS FUS mutation. He showed that the cytoplasmic localization of mutant FUS perturbs the RBP network which regulates the activities of important RBPs (HuD/ ELAVL4 and FMRP). Specifically, mutant FUS competes with FMRP for the binding of HuD 3′UTR and results in its upregulation. Consequently, NRN1 and GAP43, which are targeted by HuD, are stabilized and induce increased axon branching and growth upon injury; a phenotype which can be rescued by dampening NRN1 levels (Garone et al., 2021).

## Non-coding RNA functions and mechanisms with a focus on epitranscriptomics

The development of advanced RNA sequencing technologies improved the studies on the non-coding genome in mammals. It has been estimated that only the 1% of the genome encodes for proteins. The non-coding portion is mainly represented by RNA transcripts that have as-yet-unknown functions. Long non-coding RNAs (lncRNA) are the most abundant type of non-coding transcripts that are more than 200 nucleotides in length. They are classified in different subtypes, such as intergenic lncRNA, intronic lncRNA, bidirectional lncRNA, antisense lncRNA and enhancer RNA (eRNA). RNA polymerase II is generally responsible for transcription of lncRNAs. Similar to mRNAs, lncRNAs undergo splicing, 5′ ends capping and 3′ ends polyadenylation processes (Yang et al., 2022). LncRNAs could be potentially exploited as biomarkers or therapeutic targets because they are specifically expressed in normal tissues and deregulated in cancers and other diseases.

**Igor Ulitsky** (Weizmann Institute of Science – Rehovot, Israel) shed light on some questions regarding the functionality of lncRNA, their maturation process and evolution. Understanding whether their functions are modulated and correlated to disease progression could improve the therapeutic use of lncRNA. In particular, Ulitsky focused on the role of Silc1 lncRNA expressed during neurodegeneration. His group demonstrated that Silc1 lncRNA activates in cis the expression of Sox11, hence its depletion *in vivo* leads to a delayed regeneration in a tissue specific manner (Perry et al., 2018). In future, it will be possible to influence the expression of specific lncRNAs by CRISPR/Cas9 or ASOs-based technologies in order to improve and speed-up regeneration in case of neuronal lesions.

The group of **Joshua Mendell** (UT Southwestern Medical Center – Dallas, TX, USA) focuses research on the mechanisms underpinning the recruitment of RNA binding proteins (RBPs) by lncRNAs and their trafficking from the nucleus to the cytoplasm. Mendell highlighted the role of a lncRNA activated by DNA damage (named NORAD), a highly conserved and cytoplasmic RNA responsible for genome stability in mammals. NORAD binds Pumilio RBP and the loss of NORAD leads to Pumilio over-activation. Hence, Pumilio downstream targets (mitosis and DNA repair regulators) are repressed, causing genomic instability in mice. A genome-wide screening strategy was employed to investigate the mechanism underlying NORAD nuclear export. This new study shed light on the role of RBM33, a RBP able to mediate the nuclear transport of several transcripts included NORAD, demonstrating that RBM33 binds GC-rich elements in target transcripts. The NORAD-Pumilio pathway and the RBM33 preference for the GC-rich regions can be translated to other transcripts to shed light on the regulation of the interactions between RBPs and lncRNAs and the mechanisms for GC-rich transcript transport (Thomas et al., 2022).

It has been widely demonstrated that lncRNAs have a tissue-specific as well as stage-specific expression, and they might act as transcriptional regulators either on their transcription sites or on other sites of the genome. **Andrea Lauria** and his collaborators (University of Turin – Italy), aimed to unveil which lncRNAs bind the transcriptional coactivator protein p300. As results of a PAR-CLIP interaction screening, they identified about 120 lncRNAs actively interacting with p300 in embryonic stem cells. Among these, lncSmad7 binds the C-terminal domain of p300 and is responsible for maintaining the stem cell self-renewal. In conclusion, lncSmad7 recruits p300 in order to regulate genome regions in trans, in particular genes involved in the development (Maldotti et al., 2022).

**Agnese Loda** (PhD fellow at the European Molecular Biology Laboratory – Heidelberg, Germany) discussed the X Chromosome inactivation (XCI) from the RNA interactome point of view. As known, XCI is essential for female development, hence it becomes of great interest to investigate the mechanisms underpinning the kinetics of X-linked gene silencing, discriminating among the regulatory elements of certain genes and dissecting the spatial organization of the genome. She gave new insights into the regulation of X inactive-specific transcript (XIST), the lncRNA which acts as trigger and master regulator of XCI. The question about the link between dosage compensation and epigenetics remains open as well as the understanding of XCI escape that leads to disfunction of female development and sexual dimorphism (Loda et al., 2022).

**Pietro Carninci** (Human Technopole, Milan – Italy and Riken Institute, Japan) is a major expert on lncRNAs and their regulation. His group developed the cap analysis gene expression (CAGE) tool that has the dual goal to identify the transcription start sites (TSSs) of capped RNAs and measure transcripts expression levels (Maquat et al., 2022; Takahashi et al., 2021). This powerful tool manages to detect the promoters and enhancers of the transcripts at a single nucleotide resolution. The CAGE supported the development of the encyclopedia of DNA elements (ENCODE) and functional annotation of the mammalian genome (FANTOM) consortia. Firstly, a dataset (FANTOM5) shed light on the regulatory elements in a panel of human and mouse primary cells, cell lines and tissues. Subsequently, a FANTOM6 dataset identified the lncRNA in human primary fibroblasts and iPS cells, by using a knockdown approach. Using a novel approach, named RNA and DNA interacting complexes ligated and sequenced (RADICL-seq), it has been possible to map the interaction intercurrent between lncRNA and chromatin in the genome. This technique overcomes the limitation of previously adopted approaches, such as the high starting number of cells that can be impossible to obtain in some primary samples or the use of restriction enzyme with limited cutting sites on the genomes (Bonetti et al., 2020, 2021).

Non-coding transposable elements (TEs) or transposons represent a class of interesting transcriptional regulators, previously defined as ‘jumping genes’, due to their ability to move from one region of the genome to another. Their sequences are strongly conserved. LINEs and SINEs are one of the largest components of the family, mostly located in introns. The LINE1 family has more than 500,000 copies in the human genome. **Beatrice Bodega** (University of Milan and INGM – Milan, Italy), focuses her research on the regulation of gene expression involved in the maintenance of T-cell quiescence. The study draws the attention on the LINE1 family of transposable elements that are found in the group of genes significantly regulated during the function and exhaustion of effector T-cells. In particular, the regulation of LINE1 transcripts relies on the transcriptional factor IRF4, while Nucleolin is responsible for their maintenance in the chromosome. LINE1 family transcripts affect the deposition of H3K36me3, acting in cis on the genome region where they are. When T-cells are activated and differentiated, LINE1-transcripts are downregulated by means of splicing suppression mechanisms. Interestingly, strong accumulation of LINE1-transcripts has been found in dysfunctional T-cells, while the depletion of LINE-1 transcripts is able to restore the function of the T-cells and revert the exhaustion. These results could be exploited from a clinical perspective, where the T-cell exhaustion could be addressed modulating LINE1 transcripts with antisense oligonucleotides (Marasca et al., 2022).

Circular RNAs (circRNAs) are covalently closed circular RNA molecules, deriving from a biogenesis process where a downstream 5′ splice site of an exon is joined to an upstream 3′ splice site of the same or another exon. CircRNAs can interact with proteins and other RNAs, acting as microRNA sponges, can regulate transcription and they can also be translated in proteins. Recently, circRNAs gained attention because they have been found in biofluids and are under study as possible biomarkers for the detection of pathological states (Verduci et al., 2021). Little is known about the role of circRNAs in the regulation of mRNAs in physiological conditions or diseases. At this aim, **Manuel Beltran Nebot**, (Sapienza University – Rome, Italy) shed light on a new the interaction between circRNAs and mRNAs that can be a targetable mechanism to treat diseases. In particular, CircZNF609 is upregulated in many cancer types and its modulation reduces the tumor aggressiveness, acting as an mRNA sponge. Among the specific pairs of CircZNF609 and mRNAs, it is interesting to observe the one with CKAP5 mRNA, which encodes for a protein that regulates microtubules, involved in processes such as cell migration or the organization of the mitotic apparatus. When the interaction between circZNF609/CKAP5 mRNA was inhibited by means of small interfering RNAs (siRNAs) against circZNF60, the cells showed a higher sensitivity to drugs targeting the microtubules, such as vincristine or paclitaxel. This strategy shows an interesting potential for the control of cancer cells growth and in long term it will improve cancer treatments (Rossi et al., 2022).

**Marta Biagioli** (University of Trento – Trento, Italy) focuses her work on Huntington's disease, a genetic autosomal dominant neurodegenerative disorder, still lacking a pharmacological treatment. It has been shown that trinucleotide CAG repeat expansion in the *HTT* gene is the most frequent mutation in Huntington's disease. The triplet expansion leads to a longer sequence of glutamine tract at the amino terminus of the huntington protein, impairing its structural properties and functional activities. Biagioli described that the CAG expansion in *HTT* correlates with alterations in splicing events in neural progenitors. The work sheds light on new possible mechanisms that are dysregulated in Huntington's disease and the regulatory interactions with miRNAs and RBPs (Ayyildiz et al., 2021).

We are witnessing the emergence of the epitranscriptomics era. This field studies reversible chemical modifications of bases within an RNA molecule and is now taking medical research by storm since this post-transcriptional marks impact cell fate and development. There are more than 100 RNA modifications and recent studies demonstrate that many enzymes responsible for regulating RNA modifications could be ideal targets for cancer therapy (Barbieri and Kouzarides, 2020). **Isaia Barbieri** (University of Cambridge – Cambridge, UK) presented his group's research in Acute Myeloid Leukemia. They performed a CRISPR-Cas9 dropout screen in mouse leukemia cells and among the targets for cell proliferation they found an enzyme involved in 7-methylguanosine cap hypermethylation (TGS1). Though RNA-IP-Seq experiments in different human AML cell lines they identified more than 500 mRNA targets carrying trimethylguanosine on their cap structure that are highly enriched in cellular respiration and oxidative phosphorylation pathways. Importantly, TGS1 knockdown in acute myeloid leukemia results in the downregulation of oxidative phosphorylation (OXPHOS) with a consequent oxidative stress. Overall, Barbieri's group identified TGS1 enzyme as a master regulator of cellular oxygen metabolism in AML that could act as potential therapeutic target in leukemia.

The ribosome is a complex molecular machine no longer seen as a homogeneous entity. The concept of ‘specialized ribosomes’ capable of influencing gene expression through selective translation of specific mRNAs has gathered more and more attention in the past decade (Gay et al., 2022). **Anders H. Lund** (University of Copenhagen – Copenhagen, Denmark) presented the work of his lab and showed that rRNA modifications are a source of ribosome heterogeneity, impacting normal development and pathology (Jansson et al., 2021). Thanks to the RiboMeth-Seq technique (Krogh et al., 2017), able to simultaneously map and site-by-site quantify rRNA 2′-O-Me levels, they showed that differential rRNA 2′-O-methylation can give rise to ribosomes with specialized function. This technique allowed also to observe that ribosomes undergo coordinated changes in the 2′-O-Me patterns during brain development and during early germ layer-specific differentiation of human embryonic stem cells

**Andrea Ventura**’s lab (Sloan Kettering Institute – New York, NY, USA) is interested in how chromosomal rearrangements drive tumorigenesis. His group has used CRISPR-Cas9 technology to induce specific chromosomal rearrangements *in vivo* and *ex vivo* (Maddalo et al., 2014). Using recombinant adenoviruses expressing Sp-Cas9 and two guide RNAs (gRNAs) targeting the desired genomic breakpoints, they can induce deletions, inversions or duplications and can recapitulate *in vivo* molecular and biological properties of human cancer. Furthermore, the Ventura group aims at investigating the biological function of miRNAs in cancer. To do so they have generated a novel genetically engineered mouse strain that allows inducible and reversible disassembly of the miRISC complex and thus, temporally and spatially controls the inhibition of miRNA-mediated gene repression *in vivo* without affecting miRNA biogenesis (La Rocca et al., 2021). With this tool, they were able to investigate miRNA functions in adult tissues. They found that miRNA activity is dispensable for homeostasis of most tissues, except for heart and skeletal muscle. Furthermore, miRNA appears as essential during the process of regeneration, as observed for the intestine and the liver during response to acute damage induced by toxic agents.

SINEUPs are a new functional class of natural and synthetic antisense lncRNAs that stimulate translation of sense mRNAs with a cap-independent mechanism through an inverted SINEB2 element (Zucchelli et al., 2015). Synthetic SINEUPs could represent a tool to increase protein synthesis of potentially any gene of interest. **Bianca Pierattini** (Istituto Italiano di Tecnologia – Genova, Italy) aims at investigating the possible implication of N6-methyladenosine (m6A) modification in the molecular mechanism of SINEUP activity (Espinoza et al., 2021). She observed that SINEUP ASUchl1 (acting in a murine system), and the synthetic shorter miniSINEUP-DJ1 (human) are m6A-modified and demonstrated that their methylation is driven by METTL3 enzymatic activity. Her data, using Nanopore direct RNA sequencing (Nanopore DRS) to map m6A and METTL3 knockdown suggests the presence of a m6A-dependent step in the regulation of SINEUP lncRNAs.

Among the different RNA modifications described to date, N6-methyladenosine (m6A) is the most prevalent, conserved, and abundant internal RNA modification of mRNAs in eukaryotes. m6A functions as a critical regulator for gene expression and is dynamically and reversely controlled as an analogy to the epigenetic code formed by DNA and histone modifications (Dominissini et al., 2012). Accumulating evidence suggests that m6A greatly impacts RNA metabolism and is involved in the pathogenesis of many cancer types. **Alessandro Fatica** (Sapienza University – Rome, Italy) gave an overview of the impact of m6A modulation in chronic myeloid leukemia (CML). He showed that the m6A methyltransferase complex METTL3/METTL14 is upregulated in CML patients, and its knockdown inhibits cell proliferation. Furthermore, his group elucidated the consequences of METTL3 altered subcellular localization, showing that cytoplasmic METTL3 has a role independent from its nuclear catalytic activity. In fact, the knockdown of METTL3 in the cytoplasm affects translation in CML and he hypothesized that the translation-promoting effect on methylated mRNAs could represent a general mechanism for cells where METTL3 is localized in the cytoplasm (Ianniello et al., 2021). Moreover, Fatica demonstrated the importance of using m6A effector inhibitors that could represent an effective therapy for CML in the future.

**Ana Boskovic** (EMBL – Rome, Italy) investigates the molecular mechanisms that allow epigenetic information to be transmitted through gametes from parents to offspring in mammals. Previous work from her postdoc lab demonstrated that protein restriction of male mice modulates levels of various small RNAs in mature sperm. They showed that tRNA fragments are the most abundant small RNAs in sperm and, in particular, the 5′-end fragment of tRNA Glycine-GCC (5′tRF-GG) is consistently upregulated in response to low-protein diet in mice (Sharma et al., 2016). Ana's subsequent work showed that 5′tRF-GG acts as a repressor of genes associated with the endogenous retroelement MERVL, by promoting (hetero) chromatin compaction around MERVL promoters. Mechanistically, 5′tRF-GG supports histone biogenesis, acting on the conserved 3′UTR of histone pre-mRNAs. Correct processing of histone pre-mRNAs ensures appropriate histone supply during the S-phase, which is critical for genome repackaging after replication fork passage, as well as repression of chromatin-sensitive transposable elements (Boskovic et al., 2020). Overall, she provided evidence of a link connecting tRNA fragments as dietary sensors in sperm to chromatin changes that affect early embryonic genome output.

LncRNAs are emerging as key elements of the adaptive response mechanism, a process in which the cells adapt to a different environment by transcriptional and epigenetic rewiring. **Bianca Giuliani** (postdoctoral researcher at the Istituto Italiano di Tecnologia – Milan, Italy) focuses her research on the adaptive responses of triple-negative breast cancer (TNCB) cells. She developed a novel approach to search and identify functional lncRNA loci. This new method relies on the selection of candidate lncRNAs obtained by high-resolution transcriptional and epigenomic studies, followed by their functional characterization with CRISPR interference (CRISPRi). She was able to build a set of 620 lncRNA-TNBC candidates that were targeted in a CRISPRi library which was employed in parallel dropout screenings for different adaptive phenotypes. With this approach she identified about 100 lncRNAs involved in cancer phenotypes, including 3D growth, *in vivo* tumorigenesis or adaptation following drug treatment. Interestingly only few lncRNA loci were found as involved in unchallenged 2D proliferation, suggesting a specific role for lncRNAs in proliferation or survival upon challenging conditions. This represents a reliable approach aimed at identifying lncRNA loci that are relevant in cancer and provide new insights into the regulatory roles that lncRNAs can exert in cancer cell plasticity (Schmitt and Chang, 2016).

**Roberta Cacioppo** (University of Cambridge – Cambridge, UK) presented her work focused on mitotic kinase Aurora A (AURKA), a member of a family of mitotic serine/threonine kinases that is essential for cell proliferation. In the last few years different studies reported its kinase-independent activity in cancer cells (Naso et al., 2021). AURKA mRNA undergoes alternative polyadenylation (APA) that generates a long and a short 3′UTR structural isoform. Roberta investigated AURKA 3′UTR isoform-dependent expression in breast cancer and discovered that the short isoform expresses more protein and is translated at a higher rate. She showed that reduced translation efficiency of larger isoform is due to its selective binding to miRNA let7a. Overall, she uncovered a new isoform-dependent mechanism for oncogenic activation of AURKA and created a method based on reporter assays to accurately explore post-transcriptional and translational regulation of mRNA.

## RNA technology and therapeutics

This last session was chaired by Michela A. Denti and Stefano Gustincich. The first invited speaker was **Reuven Agami** (Netherlands Cancer Institute ­– Amsterdam, the Netherlands) who spoke about mRNA translation in cancer. Many tumors share an immune escape strategy called tryptophan degradation, where a shortage of tryptophan is generated upon interferon-γ (IFNγ) induction. In this pathway, tryptophan is transformed into kynurenine by the enzyme indoleamine 2,3-dioxygenase 1 (IDO1), which then inhibits the function of T cells, leading to immunosuppression. The Agami lab has investigated further the effects of prolonged IFNγ treatment on mRNA translation in melanoma cells by ribosome profiling. Agami's data show a global reduction in mRNA translation upon treatment and that, because of tryptophan shortage, ribosomes skip tryptophan codons by going out-of-frame and producing aberrant proteins (Bartok et al., 2021). Interestingly, Agami demonstrated also that this phenomenon is widespread across different cancer types. Using reporter vectors either in frame or out-of-frame in stress conditions, his group could confirm that frameshifting was detected in colon, lung, ovarian, and breast cancer cell types. In contrast, the healthy non-cancerous counterparts did not present any frameshifting. They named this event ‘sloppiness in mRNA translation’. Moreover, Agami's lab investigated whether sloppiness might be caused by MAPK oncogenic pathway activation, finding that RAS pathway activation stimulates sloppiness. (Champagne et al., 2021). To conclude, Agami reported his latest results about an alternative mechanism to frameshifting. His group observed that some proteins are produced in-frame during tryptophan shortage by incorporating a different amino acid. Thanks to mass spectrometry analysis, they could identify that tryptophan depletion results in tryptophan-to-phenylalanine substitutions, which are highly abundant in many different types of tumors, indicating that amino acid deprivations induce codon reassignments (Pataskar et al., 2022).

The work of **Frank Slack** (BIDMC/Harvard Medical School – Boston, MA, USA) is also focused on cancer, but from the microRNA (miRNA) perspective. Specifically, his lab works on diffuse large B-cell lymphoma (DLBCL), which accounts ∼30%–58% of all non-Hodgkin lymphomas (NHL). In this landscape, recently miR-155 emerged as a diagnostic marker as well as a therapeutic target (Eis et al., 2005; Fuertes et al., 2020) and cobomarsen, an oligonucleotide inhibitor of miR-155, decreased proliferation and induced apoptosis in cutaneous T-cell lymphoma (Seto et al., 2018). Because of these positive results, cobomarsen was tested further in a phase II clinical trial for the treatment of CTCL, mycosis fungoides subtype (MF-CTCL) (https://clinicaltrials.gov/ct2/show/study/NCT03713320). Since miR-155 was found to be overexpressed in patients with ABC-DLBCL in comparison with healthy controls, the Slack's group delivered cobomarsen to DLBCL cell lines, monitoring the binding of the drug with the endogenous miR-155, with a miR-155 biosensor. Cell proliferation and apoptosis were followed as outcome measurement of the cobomarsen treatment: *in vitro* results showed decreased proliferation and increased apoptosis of DLBCL cells. In addition, when the drug was administered *in vivo*, they observed reduced tumor growth and no adverse events, demonstrating that the drug was well tolerated (Anastasiadou et al., 2021). As result of this preclinical study, three ABC-subtype DLBCL patients were enrolled in the cobomarsen clinical trial (https://clinicaltrials.gov/ct2/show/NCT02580552). One patient experienced positive results. Despite the first positive results, the patient was discontinued from the cobomarsen clinical trial after 21 total doses of cobomarsen through five cycles, because of disease progression. Cobomarsen/anti-miR-155 targeting shows some promise in the clinic, but further refinements are necessary. Another project in the Slack's lab concerns miR-34. The miR-34 family is transcriptionally induced by p53 (Raver-Shapira et al., 2007), and these miRs act on important genes that regulate cell growth, cell cycle progression and apoptosis. Professor Slack's data demonstrated that miR-34 directly regulates expression of the PD-L1 immune checkpoint, because its overexpression reduces the levels of PD-L1. In addition, miR-34 stimulates T-cell killing of cancer cells (Anastasiadou et al., 2019). Finally, Slack talked about the potential of miR-105 as immune checkpoint inhibitor, since miR-105 represses PD-L1 mRNA, protein and surface expression levels (Miliotis and Slack, 2021).

**Danny Incarnato** (University of Groningen – Groningen, the Netherlands) focuses his research on static and dynamic RNA structure elements. The Incarnato lab’s aim is to develop strategies that overcome the technical challenges of identifying highly relevant RNA structure elements in living cells. The identification of putative functional RNA structure is of crucial importance, especially when it comes to RNA therapeutics. There are many softwares that predict the RNA structure based on free energy minimization, when the RNA sequence is given, but the biologically active structure is not necessarily the most stable. Because of this limitation, Incarnato's lab developed DRACO, an algorithm for the deconvolution of coexisting RNA conformations from mutational profiling experiments. They recently applied DRACO to SARS-CoV-2 genome, which identified some regions that fold into two mutually exclusive and different conformations (Morandi et al., 2021). Although DRACO is a very powerful tool, when combined with the conventional RNA probing tools, it cannot resolve all the conformations of the target RNA. This is why Incarnato’s group developed 2-aminopyridine-3-carboxylic acid imidazolide (2A3), a new probing technique, which is better than the gold-standards SHAPE or DMS, because it can also probe RNA *in vivo*. Their data show how 2A3 outperforms the standard method, in probing RNA *in vivo* in both bacterial and eucaryotic cells, and that RNA structure prediction is more accurate (Marinus et al., 2021). Finally, Incanato presented SHAPEwarp, an algorithm that identifies structurally similar parts in RNA molecules. This algorithm, which uses a similar approach to BLAST, was applied to analyze the genomes of both SARS and SARS-CoV-2 (CoVs), and Zika virus (ZIKV). SHAPEwarp identified eight novel conserved RNA elements, five in ZIKV and three in CoVs (Morandi et al., 2022).

**Davide Mariani** (Center for Human Technologies @IIT, Genova) talked about the impact of ALS-related FUS mutations on the subcellular RNA localization and membrane-less compartments composition. It is well known that FUS mutations are responsible for the incorrect localization and cytoplasmic aggregation of the protein, which causes toxic condensates, affecting all cellular functions (Vance et al., 2013) and, in particular, the dynamics of formation of stress granules, transient RNA-protein assemblies that cells form in response to external stimuli. He developed a new strategy to purify membrane-less compartments, including stress granules, to better dissect the role of mutant FUS versus wildtype FUS in the RNA composition in these granules. Furthermore, a new tool based on APEX2 labeling was optimized, to investigate the physiology of RNAs in ALS-like condition. Overall, their data are very promising and demonstrate that these new approaches will help in elucidating new knowledge about the subcellular landscape of RNA in neuronal cells.

**Paola Falletta** (Università Vita-Salute San Raffaele – Milan, Italy) presented her research on triple-negative breast cancer (TNBC), a tumor responsible for up to 20% of all breast cancers, known to be difficult to treat, because the high chance of relapse (Almansour, 2022). In particular, her work is focused on investigating integrated stress response (ISR), an elaborated conserved pathway that restores cellular homeostasis (Licari et al. 2021), in the context of TNBC. The data she presented demonstrate that activation of ISR in TNBR leads to alterations in mitochondrial protein synthesis. Furthermore, the ISR regulates mitochondria phenotype and bioenergetics, affecting the production of Reactive Oxygen Species (ROS) and enhancing the migratory and invasive potential of TNBC cells.

The session continued with the contribute of **Willeke Van Roon-Mom** (University of Leiden – Leiden, the Netherlands), speaker invited to hold the Riccardo Cortese Lecture, who shared the work of her group on the study of RNA as therapeutic molecule. Her lab works on Spinocerebellar ataxia type 3 (SCA3), known also as Machado Joseph disease, caused by CAG triplet expansion in exon 10 of the ATXN3 gene. This expansion causes a toxic gain of function, leading to neurodegeneration. They designed four different antisense oligonucleotides (ASOs) that were then transfected in SCA3 patient-derived fibroblasts, to induce exon 10 skipping. Three out of four ASOs did induce exon 10 skipping producing a shorter ataxin-3 transcript and truncated protein. Moreover, they tested these ASOs *in vivo*, in MJD84.2 mouse model, a transgenic mouse model containing a human ATXN3 gene with expanded CAG region. The efficacy of the ASOs was similar to what observed in SCA3 patient-derived fibroblasts. Surprisingly, the ASOs had a long-lasting effect in mice, without affecting bodyweight or motor behavior (Toonen et al., 2017). **Willeke Van Roon-Mom** is also co-director of the Dutch Center for RNA Therapeutics (DCRT - https://www.rnatherapy.nl/), a nonprofit consortium, whose mission is the development of RNA therapeutics for patients with very rare diseases. The first RNA-drug customized for one single patient was already designed and produced in 2019. Milasen was, indeed, a splice-modulating ASO drug, designed at Boston's Children Hospital for Mila, a child affected by a rare and fatal neurodegenerative condition (Kim et al., 2019). Thanks to Milasen, the patient's life quality improved: from 15 to 30 seizures per day, each lasting 1 to 2 min before starting the treatment, to between 0 and 20 less than 1-min seizures per day after the treatment, with acceptable side-effect and no safety concerns. Following the avenue opened by Milasen, the DCRT consortium aims at making the possibility to design more personalized medicines for single patients of ultrarare diseases in Europe a reality.

Antisense therapy is the interest of the second invited speaker of this session, **Adrian R. Krainer** (Cold Spring Harbor Laboratory, Harvard University – Boston, MA, USA), the father of Spinraza, without any doubt the most successful and groundbreaking ASO-based approved therapy so far. One of the present antisense projects in the Krainer lab concerns pediatric high-grade gliomas (pHGGs), one of the most malignant tumors. Among different mutations annotated, one very common in this type of tumor was found in the gene encoding for histone H3.3, where methionine replaces the lysine in position 27 (K27M), altering the epigenetic status of the cell. This event could then lead to tumorigenesis (Jones et al., 2017). Because treatments for this pathology are ineffective, the Krainer lab designed different ASOs that could knockdown *in vitro* the mutant protein, with positive results. Moreover, *in vivo* studies confirmed that the tested ASOs could improve survival and reduced tumor growth.

Another application of RNA as a therapy was given by **Giuseppina Covello** (University of Padua – Padua, Italy), that showed how U1 small nuclear RNA (snRNAs) can be modified as desired, to obtain chimeric antisense RNA to induce target-specific exon skipping with therapeutical purposes. She worked on mutations in retinitis pigmentosa GTPase-regulator (RPGR) gene that cause X-linked retinitis pigmentosa, a subtype of retinal dystrophy. An intronic mutation in intron 9, affects the splicing of RPGR gene, increasing the production of RPGR-transcripts containing the pseudoexon 9a (E9a) by a factor of ∼3.5. When E9a is present in the mature mRNA, a premature stop codon is introduced causing the production of a truncated protein. To restore the impaired gene expression, she designed some modified U1 antisense snRNAs molecules which target specifically E9a in RPGR mRNA. *In vitro* experiments demonstrated that three chimeric U1 snRNAs efficiently mediate E9a skipping, restoring RPGR transcript expression patterns (Covello et al., 2022).

The next speaker, **Gaspare La Rocca** (Memorial Sloan Kettering Cancer Center – New York, NY, USA), presented his work that describes the consequences of global inhibition of the microRNA pathway during tumor development. The approach used in this study relies on the inducible expression of a small protein, named T6B, able to interfere with the assembly of the microRNA-induced silencing complex (miRISC). In the first part of the talk, he discussed evidence that different tissues contain a diverse population of AGO complexes, mainly composed by low molecular weight RISC (LMW-RISC) and high molecular weight RISC (HMW-RISC) complexes. The latter conceivably represent the pool of miRISC engaged in target repression, and can be induced by the occurrence of mitogenic cues (La Rocca et al., 2015). Accordingly, tumors are enriched in HMW complexes, as compared to their normal tissue counterparts. By using a mouse model where T6B expression can be achieved in a tissue-specific manner, he showed that, following T6B expression, acute inhibition of the whole miRNA repertoire hinders cancer growth. His data are very promising and demonstrate that miRNA function is crucial for cancer development.

Finally, **Mattia Furlan** (Fondazione Istituto Italiano di Tecnologia – Milan, Italy) gave a talk about inference of transcriptional and post-transcriptional dynamics from sequencing experiments. Gene expression is determined by RNA synthesis, processing and degradation, each with a kinetic rate. Because transcriptional and post-transcriptional modulations leave specific footprints on premature and mature RNA temporal profile, this can be exploited to infer kinetic rates. Furlan developed INSPEcT, anovel approach based on the mathematical modeling of premature and mature RNA expression. This computational method can quantify kinetic rates from steady-state or time-course total RNA-seq data, without requiring any information on nascent transcripts (Furlan et al., 2020). Moreover, because a little experience in mathematical modelling is required to interpret INSPEcT results, Furlan and his colleagues developed INSPEcT-GUI, an interface that helps ‘wet’ researchers in the interaction with INSPEcT results and in understanding how kinetics rates influences gene expression programs (de Pretis et al., 2020).

## Future directions: direct RNA sequencing

Next generation sequencing (NGS) platforms have revolutionized molecular biology due to their exceptional throughput and accuracy, however, they are sub-optimal for specific purposes. For example, they provide short reads which typically cover only a small fraction of the original molecule, and this complicates tasks like genome assembly and transcriptome profiling. Moreover, the retro-transcription steps intrinsic in sequencing protocols introduce biases and preclude the direct sequencing of original RNA fragments preventing direct nucleotide modifications detection. Novel sequencing platforms have been developed in the last few years to overcome these limitations, one of the most relevant is the sequencing provided by Oxford Nanopore Technology (ONT). This technology allows direct DNA and RNA sequencing of full-length molecules and represents an optimal solution to the limitations of NGS platforms. Since its release, Nanopore sequencing has deeply impacted (epi)genomics and (epi)transcriptomics, and this session was dedicated to present examples of how this technology was applied across different biological contexts.

**Camilla Ugolini** (PhD candidate at Istituto Italiano di Tecnologia – Milan, Italy) used Nanopore direct RNA sequencing to investigate SARS-CoV-2 virus complexity. Direct sequencing (DRS) provides remarkable advantages for isoform detection, but DRS suffers of some limitation for the resolution of the sequences at the 5′ cap, as sequencing proceeds in the 3′ to 5′ direction and starts from the end of the transcript (typically the polyA tail). To overcome this problem, Ugolini used a new approach, termed as Nanopore ReCappable Sequencing (NRCeq). In NRCeq the native 5′ cap is replaced with a 5′ cap-linked RNA sequencing adapter, allowing to discriminate full-length, capped molecules from fragmented RNAs and truncated sequencing artefacts. With this new approach Ugolini was able to profile the repertoire of full-length, capped RNAs produced by the SARS-CoV-2 virus and, hence, to identify novel canonical and non-canonical sgRNAs. Overall, this work provides important insights into the mechanisms that regulate the transcription of SARS-CoV-2 sgRNAs (Ugolini et al., 2022) and represents a remarkable example of exploiting Nanopore sequencing for virus biology.

**Adriano Fonzino** (University of Bari – Bari, Italy) was selected to present ribonucleotides (rNMPs) detection through Nanopore DNA sequencing. The strategy used to characterize rNMPs on Nanopore sequencing is based on detecting sequencing errors and current intensity signals, revealing characteristic patterns for strands containing rNMPs. This strategy was applied on the circular ssDNA of M13 phage containing ribonucleotides at specific positions. Primers complementary to the M13 phage were designed and extended using the ssDNA as template, and the resulting double strand DNA molecules were linearized and sequenced. By means of state-of-the-art bioinformatic tools, Dr. Fonzino was able to reveal the characteristic patterns for strands containing rNMPs. This preliminary work supports the feasibility of rNMPs detection from nanopore sequencing data and suggests the possibility for interesting advancements with potential applications in the study of genome stability.

**Sìlvia Carbonell Sala** (Center for Genomic Regulation – Barcelona, Spain) from Guigó Lab tackled the problem of the characterization of the noncoding transcriptome. Since the old dogma of coding RNAs has been questioned, more and more noncoding transcripts have been discovered. However, the great majority of these RNAs (>97%) remain functionally uncharacterized. This problem is partially due to the lack of a complete annotation, with many gene models being fragmented and/or uncatalogued. As part of the GENCODE consortium, the Guigó lab recently developed a new method, capture long-read sequencing (CLS), to improve accuracy and completeness of the lncRNA catalogue (Carbonell Sala et al., 2021). This method employs oligonucleotide capture to enrich a cDNA sample for transcripts of interest, which are then sequenced with long-read RNA-seq. Although this is a great improvement, CLS still produces large proportions of 5′-incomplete transcript models. To overcome this problem, Dr. Carbonell Sala merged CLS with the CapTrap protocol (CapTrap-CLS) to enrich for 5′ capped RNAs. The libraries generated by this approach and sequenced with Nanopore and PacBio considerably improved the annotation of RNAs 5′ ends and provided one of the broadest and most accurate views of the noncoding transcriptome.

## Awards

The 2022 “*Riccardo Cortese”* prize for the best selected talk went to Marianna Maniaci (PhD candidate, European Institute of Oncology – Milan, Italy). This prize was awarded in loving memory of Professor Riccardo Cortese, a proactive member of the international scientific community who provided outstanding basic and translational results in the field of gene expression.

The 2022 “*Francesca Martini”* prize for the best poster on cancer research went to Francesca Orso (PhD candidate, University of Turin – Turin, Italy), who presented a contribute focused on the role of the miRNA miR-214 in mediating the crosstalk between stroma and malignant cells during tumor progression. This prize was awarded by NAnA onlus, a non-profit organization created in loving memory of Francesca Martini to actively support the promotion of scientific research on prevention and treatment of tumors.

Finally, the following participants were selected for the best poster awards: Chiara Barzan (PhD candidate IUSS – Pavia, Italy), Davide Colaianni (PhD candidate, University of Padua – Padua, Italy), Stefano Cretti, (PhD candidate, University of Trento – Trento, Italy), Dario Dattilo (PhD candidate, Sapienza University – Rome, Italy), Alessandro Ferrando (PhD candidate, University of Trieste – Trieste, Italy), Silvia Piscitelli (PhD candidate, University of Naples – Naples, Italy), Francesca Priante (PhD candidate, University of Turin – Turin, Italy) and Daniele Viavattene (PhD candidate, University of Turin – Turin, Italy).

## Conclusions

After two years of online conferences, the 17th annual meeting of the SIBBM has been a fresh new start for the scientific community. Overall, it represented a great opportunity to discuss about new frontiers in the RNA world. Among the many topics touched during the conference, the relevance of RNA beyond protein synthesis emerged as particularly relevant in developmental process and in many diseases including cancer and neuro-diseases. New concepts like RNA modifications, RNA structure, RNA localization and phase separation are also proving to be crucial in the field and possibly interconnected. Novel techniques, mainly for imaging and sequencing, were also introduced along with the exciting insights they might provide in all the aforementioned topics, and into coding and non-coding RNA functions across physiological and disease conditions. For a remarkable number of young speakers, the SIBBM conference has been their first opportunity to present their work in front of an outstanding audience of 270 scientists. Together with the two poster sessions, this resulted in an intense scientific discussion, palpable during the three days of conference, which will impact the future research activities on the RNA field, both in Italy and across borders.
